# Contrast-enhanced ultrasound analysis of tissue perfusion in tumor-bearing mice following treatment with endostatin combined with radiotherapy

**DOI:** 10.3892/etm.2014.1594

**Published:** 2014-02-28

**Authors:** WEI GE, YONGFA ZHENG, ZEZHANG TAO

**Affiliations:** 1 Department of Oncology, Renmin Hospital, Wuhan University, Wuhan, Hubei 430060, P.R. China; 2 Department of Otolaryngology, Renmin Hospital, Wuhan University, Wuhan, Hubei 430060, P.R. China

**Keywords:** ultrasonography, dynamic vascular model reconstruction, endostatin, radiotherapy

## Abstract

The aim of this study was to observe the effects of Endostar (recombinant human endostatin) and radiotherapy, singly or in combination, on blood flow in mouse tumour tissue using ultrasound imaging. The ultrasound contrast agent, SonoVue, was used for the contrast-enhanced ultrasound examinations. SonoLiver software was used to analyse dynamic vascular patterns (DVPs) in the contrast process. Blood perfusion data were collected and statistical analysis was performed for data processing. Results were presented as DVP curves and quantitative parameters. Quantitative parameters showed statistically significant (P<0.05) differences in peak strength, rise time, time to peak and mean transit time among the various treatment groups. Changes in tumour blood perfusion were quantified by the assessment of contrast-enhanced ultrasound parameters. The results indirectly reflected the degree of change in angiogenesis in the tumour following experimental intervention. Ultrasound contrast imaging effectively showed the extent of the changes in vascularity and flow state. Therefore, contrast-enhanced ultrasound is suitable for use as an indicator of blood flow changes in an experimental model.

## Introduction

A previous study demonstrated that Endostar (recombinant human endostatin) improves the efficacy of radiotherapy in cell and animal experiments (1). Endostar, which has a synergistic effect with radiotherapy, may act by inhibiting radiation-induced hypoxia-inducible factor-1α and vascular endothelial growth factor, as well as by upregulating aquaporin 1 expression (2). The vascular normalisation theory is widely recognised; Endostar normalises tumour vascular structure and function (3). Endostar combined with radiotherapy significantly inhibits the growth of Lewis lung cancer and may affect tumour angiogenesis. The present study aimed to investigate whether normalised vessels strengthen the role of radiation therapy of tumours by improving tumour tissue perfusion, thereby reducing the tumour hypoxia.

In the present study, the method of visualisation was contrast-enhanced ultrasonography. Intravenous injections of SonoVue, a second-generation contrast agent comprising microbubbles of sulfur hexafluoride, are widely used for abdominal and superficial organ tumour imaging (4). The advantages of using SonoVue include enhanced blood backscattering and a clearer blood flow image. The echo of the contrast agent in the blood is uniform and pseudomorphism is not easily produced (5).

The mechanical index (MI) of the ultrasound contrast agent used in the present study is <0.15, which is lower than that of microbubbles. Therefore, the energy of the ultrasound waves when reflected not only achieves continuous harmonic imaging but also reduces the disturbance of harmonics from the surrounding normal tissue (6). Perfusion in the tumour tissues of various treatment groups may be measured through low MI images (7). In the present study, this method was selected to visualize the changes in tumour blood perfusion associated with various experimental factors at different times. In addition, the targeting of tumour vascular perfusion following the administration of antiangiogenic drugs and radiotherapy was possible.

## Materials and methods

### Animals

In total, 192 specific pathogen-free female C57BL/6 mice weighing 17–20 g were used. Four of the mice had Lewis lung cancer. The diameter of the lump in the left forelimb armpit was ~1.5 cm. The mice were reared in the Experimental Animal Center of Renmin Hospital of Wuhan University (07203P; Wuhan, China). The study was carried out in strict accordance with the recommendations of the Guide for the Care and Use of Laboratory Animals of the National Institutes of Health (8th edition). The animal use protocol was reviewed and approved by the Institutional Animal Care and Use Committee of Wuhan University.

### Model and grouping

Lewis tumour-bearing mice were sacrificed by cervical dislocation on a sterile bench. Mouse tumour homogenate was stripped and cut into pieces. The living cell suspension was used for subcutaneous inoculation of the tumour. The C57BL/6 mice were subcutaneously inoculated to provide a tumour volume of 80 mm^3^. The mice were randomly divided into four groups as follows: Blank control (control), Endostar (ES), radiotherapy (RT) and Endostar combined with radiotherapy (ES + RT). For the control group, each mouse was administered a subcutaneous injection of 0.2 ml saline for 14 days. For the ES group, each mouse was administered a subcutaneous injection of 0.2 ml Endostar (Simcere Pharmaceutical Group, Nanjing, China) for 14 days. Each mouse in the RT group was subjected to 6 MeV electron irradiation with a radiation dose of 2 Gy/day for five consecutive days (source to skin distance, 100 cm) for a total dose of 10 Gy. In the ES + RT group, each mouse was administered a subcutaneous injection of 0.2 ml Endostar for 14 days and concomitantly received radiotherapy from day 1 to day 5 of the 14 days.

### Image analysis

Contrast video was stored in DICOM format and SonoLiver CAP software version 1.0 (TomTec Imaging Systems GmbH, Unterschleissheim, Germany) was used for quantitative analysis. Three regions of interest (ROI) were painted in the contrast images. Boundary, lesion and reference ROIs were coloured blue, green and yellow, respectively. The areas of depth of the lesion and reference ROIs were equal. Enhanced level differences were shown by the dynamic vascular pattern (DVP) curves and quantitative parameters. The DVP curves reflected the change process of enhancement level difference with time between the tumour and the surrounding normal tissue.

### Statistical analysis

SPSS version 17.0 statistics software (SPSS, Inc., Chicago, IL, USA) was used to analyse the data. Measurement data are expressed as mean ± SD. Single-factor analysis of variance was selected as the statistical method. P<0.05 was considered to indicate a statistically significant difference.

## Results

### Ultrasonic observations of tumour size and blood flow distribution

**Figure 1:**
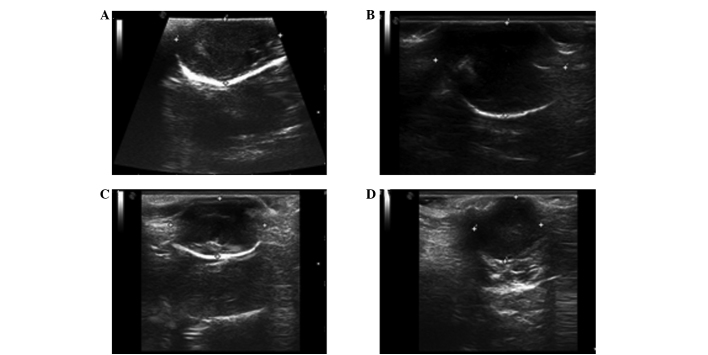
Two-dimensional ultrasound images of the tumours from rats in the (A) NC, (B) ES, (C) RT and (D) ES + RT groups. NC, control; ES, Endostar; RT, radiotherapy; ES + RT, Endostar combined with radiotherapy.

**Figure 2:**
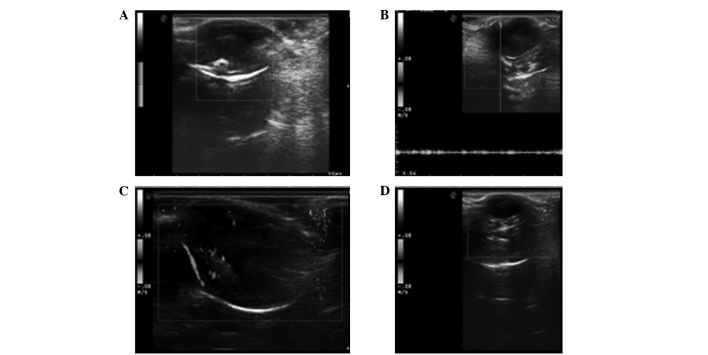
Doppler images showing blood flow changes of the tumours from the (A) NC, (B) ES, (C) RT and (D) ES + RT groups. NC, control; ES, Endostar; RT, radiotherapy; ES + RT, Endostar combined with radiotherapy.

Two-dimensional ultrasonic observations of the internal mouse tumour region showed hypoechoic and heterogeneous changes. A clear boundary interval was observed in the normal tissue around the tumour (Fig. 1). Tumour growth was observed for 14 consecutive days and colour Doppler flow imaging visualised the punctate, linear or branching irregular signal distributions around the tumour and parts of the interior region. The contrast signal was significantly increased following contrast enhancement. Blood flow perfusion and regional distribution changed as the tumour increased. The distribution of the blood flow area was not consistent in the control group. In the groups that were administered Endostar or radiotherapy alone, decreased vascular enhancement in the tumour area was observed in a circular distribution boundary. The enhanced region of blood flow in the combined group was the strongest, given that the experimental factors contributed to relatively normal tumour neovascularisation (Fig. 2).

### Analysis of the DVP curves

SonoLiver quantitative analysis software was used to reconstruct a dynamic vascular model based on the parameters of the level of difference between the tumour and the surrounding normal tissue. Each point on the DVP curve reflects the change in the level of difference with time of the enhancement between the tumour and the surrounding normal tissue. DVP curves have an automatic correction function, as they employ motion compensation to reduce or eliminate the artefacts caused by the movement of the probe.

**Figure 3:**
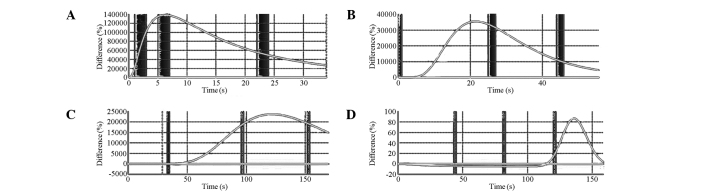
DVP curve analysis of the tumours from the (A) NC, (B) ES, (C) RT and (D) ES + RT groups. NC, control; ES, Endostar; RT, radiotherapy; ES + RT, Endostar combined with radiotherapy; DVP, dymanic vascular pattern.

Based on the relationship between the opening of the curve and trending of the DVP curve and the X axis, the DVP curve of the focus area was divided into three types: Regression, not subsidised and negative, which accounted for 63.41, 36.59 and 0%, respectively (Fig. 3).

### Quantitative parameters

**Figure 4:**
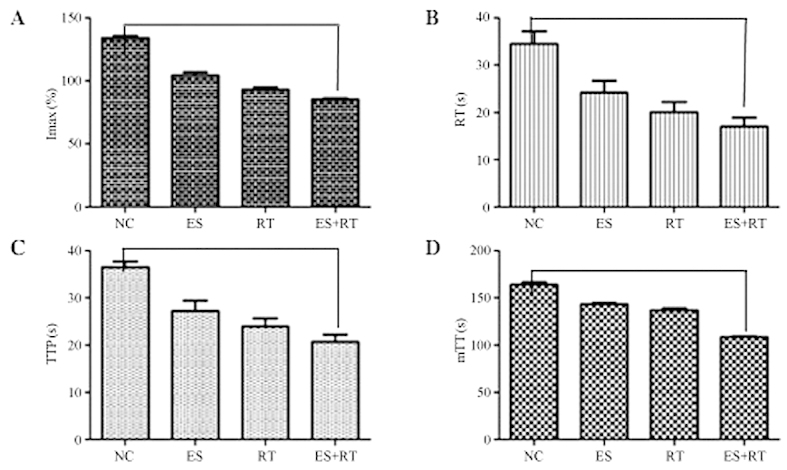
Quantitative parameter analysis of tumours in the various treatment groups. NC, control; ES, Endostar; RT, radiotherapy; ES + RT, Endostar combined with radiotherapy.

Quantitative parameters differed among the groups for peak strength, rise time, the time to peak and the mean transit time of perfusion. The differences of peak strength, rise time, time to peak and the mean transit time of perfusion in the ES+RT group and control group were statistically significant (P<0.05; Fig. 4).

## Discussion

Since the late 20th century, worldwide studies have achieved effective, quantitative blood flow imaging of experimental canine myocardium through the application of ultrasound with the analysis of parameters from microbubble refilling curves (8). The present study investigated whether it was possible to clearly observe blood flow perfusion in tumour-bearing mice using a previously described blood flow imaging system (5). By accurately determining the ROIs of the lesion, normal tissue and control area in an image using quantitative analysis software, the correct parameter measurements were ensured in the final analysis (9).

Contrast-enhanced ultrasound showed that all mice exhibited malignant tumour-specific ultrasonographic filling, which manifests as mixed masses (10). The mixed masses had uneven enhancement and their boundaries were irregular with burrs and protruding edge lengths. Two-dimensional ultrasound showed that the diameters of the tumours ranged between 1.07 and 2.16 cm, with an average of 1.37±0.9 cm. Vascular distribution patterns and scope were significantly different among treatment groups following angiography, this result was in accordance with the observation of Sirsi’s study (11). With the extension of time, the internal perfusion and peripheral tumour areas were enhanced and became more uniform in the ES + RT group. Minimal differences were observed in peripheral blood flow enhancement between the ES and RT groups, and internal flow enhancement showed a slow trend in these groups. Observations showed that the distribution of tumour vessels originated from the surrounding mass. Peripheral vessels in tumours are twisted, irregular and crab-like (12). In the absence of treatment, vascular systems exhibit a punctate appearance and are unable to achieve the effective concentration of drug perfusion and oxygen content, resulting in resistance to treatment (13). Over time, the ES and ES + RT groups showed enhanced uniformity of distribution of the tumour peripheral vascular perfusion. The interior of the tumour was more regular, with occasional sparse branches that were smooth. Tumour vascular systems became straightened with no branching between vessels. Therefore, blood perfusion in the ES + RT group was improved compared with that in the single treatment groups.

The present study used ultrasound contrast imaging to determine DVP parameters. The greyscale changes of contrast-enhanced ultrasound were transformed into colour changes and displayed in a quantitative curve form. The DVP curve provided accurate results of quantitative analysis; therefore, the results were more objective. The enhancement pattern was associated with the distribution of the microvasculature of the tumour. As the microvessel density differed among the groups, enhancement was observed at different times.

Ultrasound contrast agents are a type of blood pool contrast agent that enable the observation of the microcirculation (14). The results of the present study showed that the DVP curves of all rats in the control group exhibited rapid enhancement and fast-fading performance. In the ES group, the majority of lesions subsided, but the fading time was slower than that of the control group. In the DVP graph for the regression of tumour lesions, the extinction process curve was not uniform, which indicated that the microvessel density within the lesions differed (15). In the RT and ES + RT groups, contrast enhancement as a slow in/slow out change was observed, indicating vascular damage and suppression following radiotherapy. In the ES + RT group, the curve rose and fell rapidly at the end of the observation time, indicating that the vascular system achieved relatively effective changes in circulation following treatment with the antiangiogenic drug (13). Analysis shows that the quantitative parameters, including the peak intensity of the contrast agent in the control group, were higher than those in other treatment groups. This observation is consistent with the direct observation by contrast-enhanced ultrasound. The results of the present study show that the changes of proportion of extinction and nonresolving are apparent for intervention with different treatment factors. Moreover, the quantitative parameters of contrast-enhanced ultrasound, obtained through the analysis of DVP curves, intuitively reflect the perfusion differences between lesions and the surrounding normal tissue.

The vascular characteristics of malignant tumours are unique (16). Increased performance of the new blood vessels and lack of distribution regularity were observed. The vessel walls were not complete and defects in the basement membrane pericytes were observed. Arteriovenous shunt and vascular rings in the large tumours in the surrounding and interior regions were also observed.

Following the administration of antiangiogenic drugs, changes in vascular morphology and improved perfusion are the key points of observation. In early animal experiments, it was observed that the tumour inhibition rate in mice treated with Endostar and radiotherapy was significantly higher compared with that in the single-factor treatment groups. Moreover, the inhibitory effect on angiogenesis was the most pronounced (17–19). However, this contrasts with the increase in the overall tumour vascular perfusion.

In the present study, the DVP curves of the treatment groups varied according to different morphological changes. According to existing literature (20), this result may be associated with the improvement of the tumour vascular network. We hypothesise that although the combined treatment has the strongest effect on inhibiting the formation of blood vessels, the combined experimental factors also induce the abnormal tumour vasculature to become relatively normalised. Combined treatment may increase effective perfusion, which normalises the disorder of the vascular network, leading to sufficient oxygen and blood flow into the tumour cells. A previous study reported that Endostar in combination with radiotherapy could improve the hypoxia of the tumor cell, and change the proangiogenic factors such as VEGF (21).

Analysis of the average tumour microvessel density and microvessel count are alternative methods for evaluating tumour neovascularisation. However, these methods do not accurately reflect the efficacy of antiangiogenic therapy. Ultrasound contrast imaging accurately evaluates blood flow velocity, blood volume and blood flow parameters by mathematical models. A comparative analysis of the parameters indirectly reflects the blood flow and perfusion changes in tumours *in vivo*. This effective method may provide more quantitative perfusion information to aid treatment selection.

The present study aimed to explore the effects of an antiangiogenic therapy on tumour tissue perfusion using a low MI harmonic grey-scale contrast-enhanced method. The study produced clear images. The results enable the quantitative study of perfusion in tumour vessels and generally reflect the overall changes to the tumour during the treatment process. The study also describes an imaging method that may be used to provide information about angiogenesis and the therapeutic effects of antiangiogenic drugs (21).

Further comparison and analysis of the results with those from transmission electron microscopy and confocal microscopy are warranted, which are likely to improve understanding of the treatment effect following treatment with radiotherapy combined with antiangiogenic drugs.
